# Biosecurity and animal disease management in organic and conventional Swedish dairy herds: a questionnaire study

**DOI:** 10.1186/s13028-018-0376-6

**Published:** 2018-04-12

**Authors:** Ulf Emanuelson, Karin Sjöström, Nils Fall

**Affiliations:** Department of Clinical Sciences, Swedish University of Agricultural Sciences, P.O. Box 7054, 750 07 Uppsala, Sweden

**Keywords:** Animal disease management, Animal health, Conventional, Dairy herds, Organic, Questionnaire study

## Abstract

**Background:**

Good animal health is a notion that is germane to organic dairy production, and it is expected that such herds would pay significant attention on the health of their animals. However, it is not known if the applied animal disease management is actually more adequate in organic dairy cattle herds than in conventional dairy herds. A questionnaire study on biosecurity and animal disease management activities was therefore conducted among Swedish farmers with organic and conventional dairy cattle herds.

**Results:**

A total of 192 useable questionnaires were returned; response rates of 30.3 and 20.2% for organic and conventional farmers, respectively. Herd characteristics of the two herd types were very similar, except that pipeline/tie-stall systems were less common in organic farms and that organic farmers had a higher education level than their conventional counterparts. Also, very few systematic differences in general or specific disease management activities were observed between the two types of farms. The main exceptions being how milk from cows during antibiotic treatment was used, views on policy actions in relation to antibiotic use, and attitudes towards calling for veterinary support. Using milk from cows during antibiotic treatment was more common in conventional herds, although it was mainly given to bull calves. Farmers of organic herds were more positive to policy actions to reduce the use and need for antibiotics, and they reported waiting longer before contacting a veterinarian for calves with diarrhoea and cows with subclinical mastitis.

**Conclusions:**

The stated biosecurity and animal disease management was relatively equal in Swedish organic and conventional dairy herds. Our results thus indicate that animal health is as important in conventionally managed dairy herds in Sweden as in organically managed herds.

## Background

The European Union Animal Health Strategy 2007–2013 clearly expressed that prevention is better than cure [1]. The necessary components of such a strategy are to monitor and to identify problems, but also to take relevant actions. Prevention would seem to be even more important in organic production because organic foods are commonly marketed as being healthier foods from healthier animals. Also, European regulations on organic production state that animal health should be promoted by the use of preventive measures [2], and the Swedish Control Association for Organic Agriculture (Kontrollföreningen för ekologisk odling, KRAV) follows the EU regulations for organic dairy production and puts particular emphasis on animal health [3].

Although the production conditions in organic farms aim to promote animal health, the health situation is not always better than in conventional farms [4]. Indeed, studies on animal disease in dairy cattle herds have shown somewhat divergent results, but there has generally not been any major or consistent advantage for organic over conventional production [5–12].

The reasons for this are probably many, but little is known about how animal diseases are managed in organic herds and if such management differs between organic and conventional herds. The few studies that compare management in organic and conventional dairy cattle herds found little evidence of fundamental differences [13–18]. However, most of these studies are relatively old, and organic dairy production has undergone significant changes over the last decade. Also, several studies were performed in the US, and organic production is very different in the US and Europe. Knowing how disease is actually managed is necessary to be able to identify appropriate actions to improve animal health. There is therefore a need to identify how dairy cow diseases are managed today under European conditions.

The aim of this study was therefore to investigate Swedish organic and conventional dairy cattle farmers’ attitudes and knowledge towards biosecurity and animal disease management.

## Methods

A questionnaire was developed to acquire information on management routines that were hypothesised to be related to biosecurity and animal disease in dairy cattle herds. The questionnaire also addressed antibiotic use and resistance, but this will not be covered in the current paper. A draft questionnaire was developed based on discussions with researchers and veterinarians at the Swedish University of Agricultural Sciences (SLU), the National Veterinary Institute (SVA), Växa Sverige (the Swedish dairy association), and the staff at the food quality certification agency Kvalitetssystem Sigill AB. The draft was pre-tested on three animal science students at SLU and, after adjustments, pilot-tested on ten dairy farmers. The responses were studied to identify any confusing questions or questions that would not add any valuable information, and the questionnaire was further rephrased as needed. The final questionnaire was eight pages long, and most questions were closed, either multiple-choice or scored on a visual analogue scale (VAS).

Based on sample size calculations and practical considerations our goal was to receive 100 replies each from organic and conventional dairy farms and, considering our prior experience with distributing questionnaires to dairy farmers, a random sample of 300 organic and 500 conventional cattle farmers was drawn from the register kept by Växa Sverige.

The questionnaires were posted in the beginning of June 2014 by Växa Sverige, and the identities of the recipients were known only to them. A letter with information about the study was included with the survey, inviting the farmers to participate voluntarily. The respondents were informed that all information they provided would be treated confidentially. In September 2014, a postcard was sent to all farmers thanking them for participating in the survey and also reminding those who had not yet responded to fill in the survey and that it was possible to do so online. A note about the survey, with a link to the online version, was published at the same time in the Swedish agricultural magazine Land Lantbruk in order to increase the number of responses.

In October 2014, after the reply deadline, all data from the respondents were entered manually in Netigate (Netigate Ltd., Stockholm, Sweden). Ten surveys were randomly chosen after the data had been entered, to be used as a control of the recording process, and no errors were found. Comparisons of answers between organic and conventional farmers were performed with Kruskal–Wallis tests for replies on a continuous or and with Chi^2^ tests for categorical answers. The statistical analyses were performed using SAS (version 9.4, SAS Institute Inc., Cary, NC, USA).

## Results

A total of 198 questionnaires were returned, but four were almost completely empty and another two did not indicate if they were from organic or conventional dairy cattle farmers. The number of usable questionnaires was thus 192, of which 91 were from organic farmers. The response rates were 30.3% among the organic farmers and 20.2% among the conventional farmers. Herd characteristics of the organic and conventional farms that replied are presented in Table 1. The two types were similar in all aspects, except that organic farms used pipeline/tie-stall systems to a much lower degree and that a higher proportion of the organic farmers had post-secondary school education than the conventional farmers.

**Table 1:** Characteristics [percentage of observations for categorical variables and median (range) for continuous variables] of Swedish dairy herds participating in a questionnaire study of biosecurity and disease management in organic and conventional dairy production

Variables	Organic n = 91	Conventional n = 101	P value^a^
Age (years)	50 (20–69)	53 (23–70)	0.07
Years in profession	25 (4–45)	28 (1.5–77)	0.63
Number of milking cows	80 (40–460)	75 (25–400)	0.13
Gender of the respondent			
Female	43.3	43.6	0.97
Geographical distribution^b^			
SE1	29.7	22.0	0.24
SE21	19.8	22.0	
SE22	5.5	15.0	
SE23	27.5	22.0	
SE3	17.6	15.0	
Milking system			
Parlour/rotary	41.1	30.7	< 0.001
Automatic milking system	47.8	28.7	
Pipeline/tie-stall	11.1	40.6	
Elementary agricultural school education			
Yes	75.3	62.4	0.06
Post-secondary school education			
Yes	62.5	45.5	0.02

^a^Kruskal–Wallis/Chi^2^

^b^NUTS (Nomenclature of Territorial Units for Statistics) regions SE1 East Sweden, SE21 Småland and the islands, SE22 South Sweden, SE23 West Sweden, and SE3 North Sweden http://ec.europa.eu/eurostat/documents/345175/7451602/nuts-map-SE.pdf

Answers linked to general disease-related aspects are given in Table 2. Again, the two types were similar, except that a larger proportion of conventional farmers fed milk from cows during antibiotic treatment to calves and answered “other” for the use of such milk compared to organic farmers. The most common free-text answer explaining the “other” reply was that the milk was only fed to bull calves.

**Table 2:** Distribution (percentage) of variables linked to general disease-related aspects for Swedish dairy herds participating in a questionnaire study of biosecurity and disease management in organic and conventional dairy production

Variables	Category	Organic	Conventional	P value^a^
Herd veterinarian	Yes	52.5	68.1	0.03
Visits from livestock production advisor	At least once per month	34.1	38.6	0.66
	At least 3 times per year	24.2	21.8	
	At least once per year	24.2	16.8	
	Once every second year	7.7	8.9	
	Never	9.9	15.6	
Records kept of treated animals	Yes	91.1	83.0	0.21
	No	8.9	16.0	
	Do not know	0.0	1.0	
Hospital pen used only for sick animals	Yes	39.6	34.7	0.48
	No	60.4	65.3	
Milk from cows during antibiotic treatment^b^	Disposed of in manure	68.1	59.4	0.21^c^
	Disposed of in sewer	18.7	11.9	0.19
	Fed to calves	20.9	33.7	0.05
	Other	2.2	11.9	0.01
Milk from cows after antibiotic treatment and during the withdrawal period^b^	Disposed of in manure	39.6	36.6	0.67^c^
	Disposed of in sewer	11.0	7.9	0.47
	Fed to calves	59.3	60.4	0.88
	Other	9.9	15.8	0.22

^a^ Chi^2^

^b^ Multiple answers allowed

^c^ Comparisons between “yes” and “no” for each row

There were no differences between the two types of herds with respect to biosecurity (Table 3). Checking the climate and size of the stable was applied to a high degree in both types of herd. In contrast, there were significant differences in the respondents’ intents related to prevention of disease between the two types (Table 4). The organic farmers were especially more favourable towards policy actions to reduce the use or need of antibiotics compared to conventional farmers.

**Table 3:** Distribution (percentage) of variables linked to biosecurity for Swedish dairy herds participating in a questionnaire study of biosecurity and disease management in organic and conventional dairy production

Variables	Category	Organic	Conventional	P value^a^
Check the climate in the stable with respect to ventilation, humidity and temperature	Not done	15.4	12.0	0.28
	Might be done	22.0	32.0	
	It is done	62.6	56.0	
(Re-)Evaluate that the interior of the stable is adapted for animal size and need	Not done	8.8	10.2	0.55
	Might be done	18.7	24.5	
	It is done	72.5	65.3	
Let visitors only use clothes and shoes belonging to the farm	Not done	3.3	8.1	0.37
	Might be done	30.0	27.3	
	It is done	66.7	64.6	
Closing farm to others other than veterinarians, advisors, and family members	Not done	60.4	44.5	0.12
	Might be done	27.5	36.6	
	It is done	12.1	17.8	
Regular cleaning and disinfection of stables	Not done	0.0	1.0	0.52
	Might be done	6.6	9.0	
	It is done	93.4	90.0	

^a^ Chi^2^

**Table 4:** Respondents’ ratings of statements linked to preventive intents on a scale from 1 to 6, where 1 is “never” and 6 is “always”, from a questionnaire study of biosecurity and disease management in organic and conventional dairy production

Variables	Organic		Conventional		P value^a^
	Median (min–max)	Mean	Median (min–max)	Mean	
I want to try to prevent disease to a greater extent	6 (1–6)	5.5	6 (1–6)	5.2	0.03
If the use of antibiotics in a herd is higher than a predetermined level, a penalty and requirements for reduced usage should be required	3 (1–6)	2.9	1 (1–6)	2.0	< 0.001
Animal owners should be responsible for forming a plan together with a veterinarian on how to reduce the use of antibiotics in their herd to a certain level	4 (1–6)	3.1	3 (1–6)	2.9	< 0.001

^a^ Kruskal–Wallis

Management of a calf with diarrhoea (Table 5) or a cow considered to be at risk of having subclinical mastitis (Table 6) was also very similar between the two types of herds, with the only exception being that conventional cattle farmers tended to contact a veterinarian earlier than organic farmers; mean 2.8 and 2.3 (*P *= 0.05), respectively, where six corresponds to always and one to never contact the veterinarian immediately upon signs.

**Table 5:** Respondents’ ratings of statements linked to management of a calf showing signs of diarrhoea on a scale from 1 to 6, where 1 is “never” and 6 is “always”, from a questionnaire study of biosecurity and disease management in organic and conventional dairy production

Variables	Organic		Conventional		P value^a^
	Median (min–max)	Mean	Median (min–max)	Mean	
Isolate the calf	3 (1–6)	3.6	3 (1–6)	3.3	0.15
Check the calf’s temperature	4 (1–6)	4.3	4 (1–6)	4.2	0.73
Check the calf’s general condition	6 (2–6)	5.6	6 (4–6)	5.7	0.20
Give the calf extra comfort—blanket, more litter, or a heat lamp	4 (1–6)	4.0	4 (1–6)	4.0	0.92
Give the calf water/fluid replacement	6 (1–6)	5.5	6 (1–6)	5.5	0.98
Contact the veterinarian immediately	2 (1–5)	2.3	2 (1–5)	2.1	0.39
Wait and contact the veterinarian at the earliest after 1–2 days	3 (1–6)	3.8	3 (1–6)	3.3	0.02
Contact the veterinarian only if the calf has fever/the general condition is affected	5 (1–6)	4.7	5 (1–6)	4.6	0.68

^a^ Kruskal–Wallis

**Table 6:** Respondents’ ratings of statements linked to management of a dairy cow considered to be at risk of having subclinical mastitis on a scale from 1 to 6, where 1 is “never” and 6 is “always”, from a questionnaire study of biosecurity and disease management in organic and conventional dairy production

Variable	Organic		Conventional		P value^a^
	Median (min–max)	Mean	Median (min–max)	Mean	
Put the cow in a hospital pen	2 (1–6)	2.1	2 (1–6)	2.2	0.26
Increase cow-comfort by adding more litter/straw	3 (1–6)	2.9	3 (1–6)	3.2	0.18
Check the cell count of the milk	5 (1–6)	5.0	5 (1–6)	4.8	0.53
Send milk for bacteriological analysis	4 (1–6)	3.4	3 (1–6)	2.9	0.06
Contact the veterinarian immediately	2 (1–6)	2.3	2 (1–6)	2.8	0.05
Contact the veterinarian earliest after 1–2 days	4 (1–6)	3.5	3 (1–6)	3.4	0.64
Dry up the inflamed quarter and milk the other quarters as usual	4 (1–6)	3.5	3 (1–6)	3.2	0.12
Milk that cow separately and/or last	5 (1–6)	4.5	5 (1–6)	4.9	0.06
Milk the cow with shorter intervals	4 (1–6)	4	4 (1–6)	4.1	0.72
Milk the inflamed quarter completely	6 (2–6)	5.3	6 (1–6)	5.3	0.66
Dry off the cow earlier than planned (the whole udder) and apply dry-cow therapy	4 (1–6)	3.3	3 (1–6)	3.2	0.57
Treat the cow during the planned dry period	5 (1–6)	4.5	5 (1–6)	4.7	0.35
Dry the affected quarter if the cell count remains high after (antibiotic) treatment	5 (1–6)	4.3	5 (1–6)	4.4	0.76
Do not inseminate the cow and cull it at the next planned dry off	4 (1–6)	3.9	4 (1–6)	3.9	0.66
Cull the cow immediately	2 (1–5)	2.1	2 (1–5)	2.2	0.47
Cull the cow if an (antibiotic) treatment does not work	4 (1–6)	4.0	5 (1–6)	4.3	0.11
Cull the cow if the cell count remains high after (antibiotic) treatment	5 (1–6)	4.5	5 (1–6)	4.6	0.59
Check cell count of the whole herd	5 (1–6)	4.9	6 (1–6)	5.1	0.19

^a^ Kruskal–Wallis

## Discussion

There were no major differences in disease-related management activities between organic and conventional dairy cattle farms in Sweden. This is consistent with our previous results where we have shown that there is little difference in disease occurrence between organic and conventional dairy cattle herds in Sweden [7–10, 12]. Our results thus indicate that animal health and disease management may be just as important in conventionally managed as in organically managed dairy cattle herds in Sweden. These results also agree with other studies comparing management in the two production systems in Europe and the USA [13–18], indicating that this conclusion is not limited to Swedish conditions. The very small systematic differences between, but relatively large variation within, both groups indicate that management choices are more farm specific than dependent on the organic or conventional status of the herds.

Actions related to calves with diarrhoea or cows with subclinical mastitis were scored almost the same for the two types of herds, although organic herds tended to wait a bit longer before contacting a veterinarian. A dedicated herd veterinarian was also less common in organic herds. These results are consistent with previous studies showing that Norwegian organic dairy cattle farmers feel less in need of veterinary advisory services than conventional farmers [15]. Delayed antibiotic treatment of cows with mastitis might not be positive with respect to treatment effects and might be detrimental to animal welfare, and such delays have been reported to be a concern among practicing veterinarians [19, 20]. A contact with a veterinarian does not necessarily lead to a treatment decision, but in such cases a delayed contact will also lead to a delayed treatment because only veterinarians are allowed to initiate an antibiotic treatment in Sweden. We are not aware of any studies comparing treatment efficiencies between organic and conventional farms, but the on-farm mortality was found to be lower for organic than for conventional Swedish farms [21]. Because on-farm mortality might be a consequence of delayed veterinary treatment of diseases, the differences in actions we observed in this study might not have a negative consequence for animal health. Thus, the different use of veterinarians in organic herds might be balanced by more active handling of health disorders among their cattle by the farmers themselves, as was observed by Valle et al. [15].

The organic farmers indicated a stronger intention to prevent diseases and were more positive towards implementing policy measures to reduce the use of antibiotics than conventional farmers, although it should be noted that the readiness was relatively low on both farm types. The difference might be a result of the stronger requirements of Swedish Control Association for Organic Agriculture and the realisation among organic farmers of the value and feasibility of reducing antibiotic use. The response rate was, however, higher among the organic farmers, which might indicate higher levels of engagement in animal health issues. We also observed a significantly higher education level among the organic farmers, which might indicate a selection bias towards more well educated and interested farmers, leading to a higher awareness of preventive actions and acceptance of policy measures. However, the differences in attitudes might also be a true difference caused by the greater personal involvement that organic dairy cattle production requires.

Our results show that feeding waste milk, i.e. milk from cows not permitted for human consumption due to antibiotic treatment, to calves was more common in conventional than in organic dairy cattle herds. This was especially pronounced considering the “other” use, which was mainly indicated as such milk being fed only to bull calves. This use agrees with general observations by Duse et al. [22], and it is therefore possible that antibiotic resistance is more common in conventional dairy cattle farms because feeding this kind of milk, even just to bull calves, is associated with increased occurrence of antimicrobial resistant bacteria in the herd [23]. Future research in this regard is warranted because lower antibiotic resistance in organic dairy cattle herds would indicate a competitive advantage for organic production. It is important to realize that also other ways to dispose of waste milk may impose increased risks for development of antimicrobial resistance, also in the environment, and any use of antibiotics should therefore be well justified.

The questionnaire study was anonymous and the distribution was not under our control, so we could not perform a formal non-response analysis. However, our sample agrees with official statistics with respect to age, gender, herd size, and geographical distribution [24], and we therefore consider it reasonably representative of the Swedish dairy cattle farmer community. It is possible, however, that the responders were more positive towards animal health and disease management than the general population of dairy farmers because answering the questionnaire required some interest in the question. A questionnaire study may also suffer from response bias, where replies are not completely truthful, and we cannot verify that the answers correspond to actual actions. The limitations of studies such as this should be kept in mind when interpreting the results, but there is no reason to believe that possible biases would be differential, i.e. invalidate the comparisons between organic and conventional farmers.

There were no major differences in the general characteristics of the herds in the sample, except that tie-stalls were more common in conventional herds and that automatic milking systems (AMS) were more common in organic herds. This is probably rather unique to the Swedish situation because a comparison between organic dairy farms in France, Germany, Spain, and Sweden showed that AMS was rarely used in these other countries [25]. However, tie-stalls were also found to be more common among equally sized conventional herds than organic herds in the US [18], but the extent of AMS systems in that sample is not known. We have previously shown that there are differences in implemented mastitis management options between Swedish dairy farms with and without AMS [26], so the differences in milking systems might impose differences in management between the two types of herds. However, few of the questions in this study were directed to mastitis management, and any such differences are thus not likely to have influenced the comparisons to any significant degree.

## Conclusions

The stated biosecurity and animal disease management was relatively similar in Swedish organic and conventional dairy cattle herds, although farmer attitude towards health improvement might be somewhat different. Future research should study if the different use of veterinarians might have an impact on treatment success and if the different use of waste milk might have an impact on antibiotic resistance in these types of herds.
